# Psychosis breakthrough on antipsychotic maintenance medication (BAMM): what can we learn?

**DOI:** 10.1038/s41537-017-0039-z

**Published:** 2017-10-11

**Authors:** Jose M. Rubio, John M. Kane

**Affiliations:** 1 The Zucker Hillside Hospital, Psychiatry Research, Northwell Health, Glen Oaks, NY USA; 2 Hofstra Northwell School of Medicine, Hempstead, NY USA; 3 The Feinstein Institute for Medical Research, Manhasset, NY USA

Antipsychotic drugs are highly effective in preventing relapse among individuals with schizophrenia, with a number needed to treat of 3.^1^ Suboptimal adherence with antipsychotic treatment is by far the greatest predictor of relapse in psychosis,^2^ yet a subset of patients relapse despite full adherence with antipsychotic drugs. Psychosis Breakthrough on Antipsychotic Maintenance Medication (BAMM) represents a barrier to the successful maintenance treatment of schizophrenia, yet it has received surprisingly little attention. We believe that a better understanding of this phenomenon could have important implications for the field.

Treatment adherence is a challenge in any chronic illness and is very often suboptimal over time in patients with schizophrenia.^3^ Given the lack of reliable and practical methods to measure actual exposure to antipsychotics,^4^ research on the effects of oral antipsychotics is potentially confounded by poor/partial adherence. We would emphasize the potential value of studying BAMM in individuals treated with long-acting injectable (LAI) antipsychotics, where adherence can be confirmed by the dates of treatment administration. To our knowledge, the literature on BAMM in such individuals is limited to a post-hoc, secondary analyses of one clinical trial.^5^ In this recent study, Alphs and colleagues found that 18.3% of patients with schizophrenia who were stable at randomization and treated with LAI risperidone had a relapse within 1 year of treatment. They identified illness duration and clinical research site as independent predictors of BAMM. While this study has provided relevant initial data, these results should be replicated and the role of clinical, epidemiological and neurobiological variables in BAMM should be investigated.

Why would a treatment to which there was once a clinical response stop being efficacious? The mechanisms underlying this phenomenon are far from clear and have received remarkably little attention. Animal models involving chronic exposure to antipsychotics indicate a “breakthrough” of dopaminergic activity despite antipsychotic treatment due to an increased expression of number and affinity of dopaminergic receptors over time.^6^ Whether this occurs in humans is not known,^7^ though tardive dyskinesia has been attributed by some to such a phenomenon.^8^ It has also been hypothesized that response to antipsychotic drugs could serve as a phenotype for distinct underlying pathophysiology, with treatment responsive schizophrenia being more associated with hyperdopaminergic activity, and treatment resistant illness being normodopaminergic.^9^ Whether hyperdopaminergic activity would be observed in patients with BAMM, who were once presumably responsive to treatment, yet subsequently relapse despite antipsychotic maintenance medication, has not been studied either. Whether dysfunctions in systems other than the dopaminergic, (i.e., glutamatergic) or risk factors for relapse other than non-adherence (i.e., comorbid substance use) are involved in BAMM is also unknown. The study of BAMM in patients receiving LAIs could serve to address these and other related questions in a clinical model not confounded by non-adherence.

We anticipate that a better understanding of such mechanisms could have implications for other areas in the field, ranging from the development of more efficacious relapse prevention strategies to a better understanding of the underlying pathophysiology of schizophrenia, or schizophrenia subtypes. For instance, the identification of epidemiological predictors could serve in the development of early intervention for individuals at risk of BAMM, and the identification of neurobiological correlates could help to develop biological treatments that minimize relapse. Research on BAMM could also help to clarify key questions about the efficacy of antipsychotic medications that remain unclear because of the potential confound of non-adherence. The study of the efficacy of antipsychotic drugs in individuals who stay adherent and do not discontinue treatment has been limited. Obtaining reliable measurements of the incidence of BAMM could provide additional information about the efficacy of antipsychotic medications. This could contribute to the current debate about the overall effectiveness of these drugs, which six decades after their development is still being questioned.^10^ Another circumstance that could be clarified by further study of BAMM is the apparent decrease in efficacy of medication over time in long term studies.^1,11^ Whether this is due to a cumulative effect of non-adherence or to an actual decrement in the effect of antipsychotics is unclear. Understanding the role of time on treatment in the risk for BAMM could clarify this important question. Research on BAMM could also help to elucidate the role of risk factors for relapse in schizophrenia. For example, a recent study found that the effect of cannabis on psychotic relapse may be partly mediated through suboptimal adherence with antipsychotic treatment.^12^ Studying the role of substance use in BAMM could shed light on the association between cannabis use and risk for relapse in psychosis.

Of special importance is the relationship between BAMM and treatment resistance. Despite decades of research, the mechanism(s) underlying treatment resistance remain elusive.^13^ With as many as one third of patients developing treatment resistance,^14^ and clozapine, with a burdensome tolerability profile, having been the only drug approved for this indication in twenty seven years,^14^ it is urgent to understand its pathophysiology and to develop alternative treatments. BAMM could serve as a clinically valid paradigm to move forward in this area. We would hypothesize that the failure to maintain treatment response in BAMM could share elements with treatment resistance, and that the understanding of the pathophysiology of BAMM could help us to better understand treatment resistance. We need to test this hypothesis, as it is also possible that BAMM represents a different phenomenon from the inability of antipsychotics to reduce existing psychotic symptoms to a clinically meaningful degree. Future research in this area should utilize BAMM as a paradigm for the potential validation of emerging biomarkers of treatment response and failure.^9,15^

In sum, BAMM represents a clinically meaningful phenomenon with important treatment implications that has received little attention. Converging research from the perspectives of clinical trials, epidemiology and neurobiology in this area has the potential of not only advancing relapse prevention, but also informing other areas of important uncertainty.
